# Supplementary material for: Learning curve and surgical outcome of robotic assisted colorectal surgery with ERAS program
Source: Sci Rep. 2022 Nov 29;12:20566. doi: 10.1038/s41598-022-24665-w (PMC9709162; doi:10.1038/s41598-022-24665-w)
Supplement: Supplementary file 2 — Supplementary Information 2. [file 41598_2022_24665_MOESM2_ESM.zip › RAL Raw data-Ñ[▒K/123/HS_ñw╝╨░O▒Kñσ.pdf]

正式摘要(電子簽章：000000229581770) W83 / W83 ☐英文標題(注意:無英文姓名-請補病人基本檔!)	☐【讀取】憑證

臺中榮民總醫院

出院病歷摘要

轉入號： 轉出號：

[redacted]

**(8)**流水編號	
**(9)**入院日期	110/10/28 (2021) 直腸外科 W83 - 066
**(10)**轉科日期	
**(11)**出院日期	110/11/10 (2021) 住院天數計 13 日

診斷

- 入院 :Adhesion ileus
- 出院 :Adhesive ileus on 2021/10/12-24 with recurrence since 2021/10/27
  - s/p Robot Assisted Adhesionlysis on 2021/11/02
  [PHx]
  1. Prostate carcinoma in situ s/p surgery in 2007
  2. Type 2 Diabetes Mellitus, under medication contol
  3. Hypertension, baseline SBP around 150mmHg, under medication contol

主訴

- acute cramping pain with vomiting at 10/27 midnight

病史

- This is a 76-year-old male patient with past history of
  1. Prostate carcinoma in situ s/p robotic surgery in 2007
  2. Type 2 Diabetes Mellitus, on medication with Vildagliptin (Glavus 50mg) 1# BID, Grumed (Glimepiride 2mg, =Amaryl in VGHTC) 1# BID, Acarbose (Carlipin 50mg) 1# BID
  3. Hypertension, baseline SBP around 150mmHg, on medication with Cozaar
  He denied ABC(-).

  Last admission, he had same condition during 10/12-10/24. Due to smooth flatulence and stool passage, he was discharged on 10/24. Red flag signs were told.

  However, he had acute cramping pain with vomiting at 10/27 midnight. Thus, he went to our ER for help. During our ER, his vital sign were BP=182/107, HR=115,RR=20, BT=36.2. Abdominal PE showed distant, tympanic, hypoactive bowel sound, tenderness over A5, no muscle guarding, no rebounding tenderness. KUB showed bowel dilatation with gas and multiple air fluid level noted at upper abdomen. Lab revealed leukocytosis with neutrophil predominant, mild hyponatremia, and elevated creatinine. Due to above evidence and previous CT scan, adhesive ileus was impressed. We prescribed cefa+mentro as empirical antibiotics. Moreover, we keep NPO with NG decompression and hydration.

  There was no fever/chillness, no SOB, no chest pain, no diarrhea, no stool/flatus passage, no other specific discomfort.

  With the impression of adhesive ileus, he was admitted for supportive care and possible surgical intervention.

體檢發現

- 2021/10/12 CT: small bowel loop.
  - General appearance :
    - easy looking , conscious clear , without cardiopulmonary distress
    - Vital signs自動帶入date 體溫(BT) 脈搏(P) 呼吸(R) 血壓(BP) 身高(BH) 體重(BW) BMI
      20211028 35.7℃ 78次/分 18次/分 160/72mmHg 167.4cm 67.1kg 23.9
    - Vital signs: B.P. 160/72 mmHg , P.R. 78 /min , R.R. 18 /min , B.T. 35.7 ℃
  - HEENT :
    - Eyes: no anemic conjunctiva, no icteric sclera, normal EOM
    - ENT: no otorrhea, no rhinorrhea, no erythematous mucosa, no injected tonsil, no deformity
  - Neck: supple, freely movable, trachea midline,no deformity, no engorged jugular vein , no palpable thyroid or lymph node enlargement
  - Chest:
    - Inspection: Grossly normal, symmetric and fully expansion, no accessory muscle used, no operation scar, no spider angiomata, no gynecomastia
    - Palpation: no palpable mass or nodule, no subcutaneous emphysema, no tenderness
    - Ausculation: clear breathing sound
  - Heart:
    - Percussion: normal heart size
    - Ausculation: regular heart beat, normal S1, S2, no gallop, no murmur
  - Abdomen:
    - Inspection:
      - distension: + , operation scar: previous robotic scar
    - Ausculation:
      - hypoactive bowel sound
    - palpation: soft, no muscle guarding or rigidity, no local tenderness, no rebounding pain, no palpable mass, no palpable pulsation
    - Percussion: no shifting dulllness, no hepatomegaly, no splenomagaly
  -

Peripheral pulsation :	CA	BA	RA	FA	PA	PTA	DPA
Right side :	++	++	++	++	++	++	++/+++
Left side :	++	++	++	++	++	++	++/+++

  - Genito-anus: no tenderness or palpable mass lesion or rectal shelf, normal external genitalia, normal anal tone, brown stool

CRS CONDITION

  - Imaging studies(Sono , CT of abdomen and chest Films):
    - 檢驗項目 :ABDOMEN (KUB),SUPINE 報告日期 :1101028
      主 訴：
      Result:N Finding: > Much bowel gas and bowel dilatation. > Bowel obstruction can not be excluded.
      Impression: As above description.
      檢驗項目 :ABDOMEN (KUB),SUPINE 報告日期 :1101028
      主 訴：
      Result:N Finding: Much bowel gas and bowel dilatation. Bowel obstruction was highly suspected.
      Recommend closely follow-up. Impression: As above description.
      檢驗項目 :ABDOMEN(KUB),STADING 報告日期 :1101028
      主 訴：
      Result:N Finding: > Bowel dilatation with gas and multiple air fluid level noted at upper abdomen.
      Bowel obstruction was highly suspected. Impression: As above description.
  -
    - Lung

手術日期及方法

- 手術日期及方法20211102 Robot Assisted Colectomy(L)

住院治療經過

- After admission, we kept following his KUB which revealed still bowel dilatation with gaseous movement. Thus, we removed his NG tube and allowed him to try water. PN supplementation also was given. Due to recurrent of ileus, we suggested him undergoing the operation. After discussing the pros and cons of the operation, he decided to receive the robotic surgery. Robot

- Assisted Adhesionlysis was performed on 2021/11/02. Post op care including abx, pain control and symptomatic tx was given. He had stool and flatus. The diet was titrated up step by step and he tolerated well and PN was then off. Abdominal wound recover well with the suture was removed. Under relative stable condition, he was discharged with OPD f/u.

合併症

- nil

一般檢查

- 累積報告：

DATE	TIME	ALB	TP	BIL.T	BIL.D	ALKP	AST	ALT	LDH	AMY	LIPASE	NH3	Ferritin	UA
1101027	1825						34	30						
1101028	1625	3.7												
1101101	0905			0.35				13						
1101101	1241	3.6												
1101102	1830			0.72			29	14						
1101105	0856	3.3		0.5	0.2		29	10						
1101110	0858			0.67			25	9						

DATE	TIME	WBC	RBC	HGB	HCT	MCV	PLT	BLST	NEUT	BAND	SEG	LYM	MON	EOS	BAS
1101027	1824	15220		15.9			353		86.6			8.4	4.7	0.1	0.2
1101101	0931	4000		13.8			277		62.4			25.5	9.8	1.8	0.5
1101102	1830	11670		15.5			312		82.1			13.3	4.2	0.3	0.1
1101105	0855	4940	4.42	13.2	37.8	85.5	232		72.3			18.0	7.9	1.6	0.2
1101110	0904	8240		14.3			315		69.9			18.0	7.6	4.0	0.5

DATE	TIME	NA	K	CL	BUN	CREAT	eGFR	CA	Mg	P	CRP	ESR	PCT	D-Dimer	LACTATE
1101027	1825	133	4.0	98	24	1.37	53.69	9.3							
1101028	1625								2.3	2.4					
1101101	0905	138	3.7		7	0.88	89.49	7.5							
1101102	1830	134	3.5		6	0.69	118.49	8.2							
1101102	1841														17.5
1101105	0856	133	3.4		9	0.78	102.86	7.5							
1101110	0858	136	3.6		13	0.88	89.49	8.4							

DATE	TIME	GLUCOSE	HbA1C	CHOL	TG	HDL	LDL	KETONE	CK	CKMB	TRO-T	PT-P	APTT-P	NT-pro BNP
1101027	1825	197										10.6	26.6	
1101028	1625			126	37									
1101101	0926		7.1											

DATE	TIME	PH	BEB	SCVO2	PO2	HCO3	HGB	PCO2	TCO2
1101102	1829	7.416	0.0	99.3	131.4	24.3		38.7	25.5

- 放射線報告：檢驗項目 **:CT ABDOMEN-SURVEY** 報告日期 **:1101101**
  上 訴：
  Result:Y
  Finding:
  **Contrast enhanced CT scans were performed from liver dome to pubic symphysis .
  SHOWED:
  Tiny cal. at S7 of liver.
  Mild bowel dilatation with mild wall thickness and some fluid retention found at distal ileum. No definite bowel obstruction.
  No other specific findings noted at liver, spleen, pancreas and kidneys.
  The lower abdomen showed no significant unusual.
  No bulky lymphadenopathy noted.
  Impression:
  Mild bowel dilatation with mild wall thickness and some fluid retention found at distal ileum. No definite bowel obstruction.
  For other details, please see the above descriptions.

特殊檢查

病理報告

- nil

其它

出院時情況

- 3.改門診治療

出院指示

藥名	劑量	劑量單位	用法	頻次	數量	數量單位	現況	天數	單位
MOSAD tab 5mg	1	TAB	PO	TID	21	TAB	TH	7	天
Ultracet tab	1	TAB	PO	Q6H	28	TAB	TH	7	天
Econ tab 40mg	1	TAB	PO	QID	28	TAB	TH	7	天

出院計劃

- 門診日期
  - date on 2021/11/16
- 門診追蹤。

主治醫師 2222H 林俊余 / 住院醫師 2351A 林易菁

正式摘要(電子簽章：**000000229581770)**

W83 / W83

doctorRank:B
